# Antimicrobial effectiveness of Nano Silver Fluoride Varnish in reducing *Streptococcus mutans* in saliva and plaque biofilm when compared with Chlorhexidine and Sodium Fluoride Varnishes

**DOI:** 10.4317/jced.59093

**Published:** 2022-04-01

**Authors:** Nandita Waikhom, Nidhi Agarwal, Zohra Jabin, Ashish Anand

**Affiliations:** 1 Post Graduate student, Department of Pediatric Dentistry, Institute of Dental Studies and Technologies, Modinagar; 2 Professor and Head, Department of Pediatric Dentistry, Institute of Dental Studies and Technologies, Modinagar; 3 Professor, Department of Pediatric Dentistry, Institute of Dental Studies and Technologies, Modinagar; 4 Reader, Department of Pediatric Dentistry, Institute of Dental Studies and Technologies, Modinagar

## Abstract

**Background:**

This *in vivo* study was done to investigate the antimicrobial effectiveness of Nano Silver fluoride, Sodium fluoride and Chlorhexidine when used as a varnish on *Streptococcus mutans* (*S.mutans*) in saliva and plaque biofilm.

**Material and Methods:**

120 caries free subjects, aged 8-10 years were randomly assigned to four different groups (n=30) - group I: Control, group II: Chlorhexidine varnish (CHX), group III: Sodium fluoride varnish (NaF), group IV: Nano Silver fluoride varnish (NSF). Varnish application was done once at baseline. Saliva and plaque samples were collected at baseline (T0), at the end of 1 month (T1) and 3 months (T3) to evaluate *S.mutans* levels by culture method, optical density and PCR. OHI-S Index was also recorded for clinical evaluation.

**Results:**

NSF, CHX and NaF were effective against *S.mutans* activity. The intragroup comparision of CFU/ml and OD/ml count showed a highly significant reduction from baseline to 3 months for all the 3 varnish groups (*p*=0.001). PCR result revealed that maximum reduction was seen in NSF and CHX followed by NaF group.

**Conclusions:**

NSF reduces *S.mutans* level in both saliva and plaque biofilm and it is more advantageous than CHX and NaF as it has dual properties of acting as an antibacterial as well as a remineralizing agent.

** Key words:**Chlorhexidine, Nano Silver fluoride, Sodium fluoride, S. mutans, varnish.

## Introduction

In 2017, The Global Burden of Disease Study estimated that about 2.3 billion people suffer from caries of permanent teeth and about 530 million children suffer from caries of primary teeth (1). Dental caries is a multifactorial disease and one of the factors responsible for caries is the oral microbial flora. Among over 600 identiﬁable bacterial species in the oral cavity, mutans Streptococci (MS) has been seen to be most closely associated with the development of dental caries (2,3). Prevention of caries is possible through reduction in the level of *S.mutans* and various agents like fluorides, chemoprophylactic or herbal medicaments are available for the same. Use of fluoride varnishes is a popular and cost effective professional method of caries prevention as it adheres to the tooth surface for longer period and exerts a long-term prophylactic effect (4).

One of the oldest and most commonly used fluoride varnishes is Sodium fluoride (NaF) that has been shown to result in significant suppression of *S. mutans* when applied on the tooth surface. Its major drawback is that it requires multiple applications (5,6) Chlorhexidine (CHX), a cationic bis- biguanide, is a broad spectrum anti-microbial agent (7) used in dentistry that has proven its effectiveness in reducing the oral mutans Streptococci count (8-11). It is available as a varnish but it has a bitter taste, high cost and esthetic issues when used in anterior teeth (12).

With the advancement in nanotechnology, research has lead to the development of Silver nanoparticles (AgNPs). Silver nanoparticles contain 20 to 15000 silver atoms having a diameter smaller than 100 nm. They have a large surface-to-volume ratio due to which they have been shown to have a remarkable antimicrobial activity, even at a low concentration. They are cost effective, show low cytotoxicity and immunological response. Therefore, silver nanoparticles have found multiple potential biomedical applications like drug delivery devices, medical imaging, molecular diagnostics, incorporated in surgical mesh, fabrication of artificial joint replacements, wound dressing and medicament for promotion of wound healing. In dentistry, silver nanoparticles are used in fabrication of dentures, composite resins, irrigating solution and obturation material, adhesive materials in orthodontic treatment, membrane for guided tissue regeneration, and titanium coating in dental implant treatment (13). Haghgoo R *et al*. in 2014 suggested the incorporation of Nano-silver in varnishes and recommended it for application for prevention of dental biofilm formation (14). In 2014, Targino *et al*. prepared a formula containing AgNP, chitosan and fluoride and termed it as Nano Silver Fluoride (NSF). This new formulation demonstrated to be a good antimicrobial agent that could inhibit *S.mutans* growth (15,16). Also, when NSF was compared with chlorhexidine and silver diamine fluoride, it was found to be both bacteriostatic and bactericidal (15). Besinis *et al*. and Hernandez *et al*. have observed AgNPs to be 25 fold higher in antibacterial activity than chlorhexidine (17,18). It has been found to have larger bactericidal activity (19), non-toxic and safe for human use (20), with a potential advantage of not staining the teeth (15). A review of available literature revealed that a majority of the studies conducted on the effects of NSF were invitro (16,21,22). Thus, the present *in vivo* study was done to observe the effect of NSF varnish on oral *S.mutans* count and to compare it with sodium fluoride & chlorhexidine varnish.

## Material and Methods

-Study design:

The triple blinded randomized parallel group trial was conducted in accordance with the CONSORT 2010 guidelines. The sample consisted of children between 8-10 years fulfilling the following criteria.

-Inclusion criteria:

• Subjects with no clinical signs of carious lesion (white spot/ cavitated)

• Subjects with the following fully erupted permanent teeth: permanent maxillary right central incisor, permanent mandibular left central incisor, all permanent maxillary and mandibular first molars.

-Exclusion criteria:

• Children with any intraoral pathology 

• Children with medically compromising, physically or mentally challenged condition.

• Antibiotic intake one month prior to the study.

• Uses of any fluoridated toothpaste or antimicrobial mouthrinse during the course of the study.

The sample size estimation was done using G Power 3.1. The desired statistical power was set at 80% (1-β), to detect statistical significance of 5% (α= 0.05) and effect size (f) 0.39. 27 subjects in each group were required to avoid any statistical type 1 errors. The sample size was rounded off to 30 in each group to cover for any loss of subjects during the follow up period. SNOSE (Sequentially numbered, opaque, sealed envelopes) method was performed for allocation concealment. To achieve allocation concealment, the random concealment was done by an investigator who was neither involved in the application of the varnish nor measurement of outcome. A piece of paper comprising of a randomized group number was sealed in the dark coloured envelope containing respective serial number over it. The envelope was opened once the intervention was assigned. Based on the group assigned in the paper, varnish application was done. 290 children were examined out of which 120 children were included in the study based on the selection criteria.

The purpose and procedure of the study was explained to the parents/ or guardians prior to commencement of the study and a written informed consent was obtained. Each child’s personal, medical and dental details were obtained and recorded.

The selected study participants were randomly assigned to 1 of 4 groups (30 children per group):

1. Group I - Control group. Saline was used as a placebo control;

2. Group II - Chlorhexidine varnish (cervitec plus by Ivoclar Vivadent, Schaan, Liechtenstein) group which includes 1% chlorhexidine diacetate and 1% thymol as active antimicrobial ingredients;

3. Group III - Sodium Fluoride (Profluoride Varnish, VOCO GmbH. Cuxhaven, Germany) Varnish group that contains 5% NaF with xylitol;

4. Group IV - Nano silver fluoride group. A special formula as prepared by Targino *et al*. (15) as given below. It contains nano silver particles, sodium fluoride and chitosan as a stabilizer.

-Preparation of Nano Silver Fluoride:

Nano Silver Fluoride was prepared according to the formulation given by Targino *et al*. (15). The synthesis of silver nanoparticles in an aqueous solution was carried out via the chemical reduction of silver nitrate (AgNO3) with sodium borohydride (NaBH4) and chitosan biopolymer as a stabilizing agent. Chitosan (2.5 mg/ml) was dissolved in a 1% acetic acid solution. The solution was mixed under magnetic stirring until homogeneous. Next, the mixture was transferred to an ice-cold bath, and freshly prepared NaBH4 (0.3 mL, 0.8 M) was then added drop by drop while stirring vigorously. The ﬂask was then removed from the ice bath and the sodium ﬂuoride (10,147 ppm of ﬂuorine) was incorporated. The stirring was maintained overnight. The prepared solution contains silver nanoparticles 399.33µg/ml, sodium fluoride 10,147µg/ml and chitosan 2334µg/ml.

**Figure 1:**
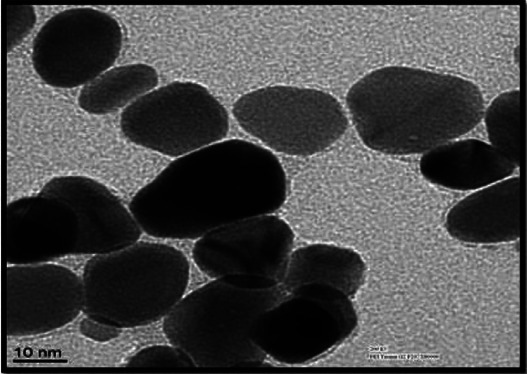
TEM image of Silver Nanoparticles used in the study.

The size and shape of silver nanoparticles were evaluated with transmission electron microscopy (TEM). TEM image were obtained on a FEI-Tecnai G2 F20 at an accelerating voltage of 200 Kv. The analysis revealed 99% of silver nanoparticles were spherical with a particle size of 8 ± 2.0 nm (Fig. 1).

Prior to commencement of the study, Child’s personal details, details of past medical history including any recent antibiotic exposure, past dental history were recorded. The study purpose & procedure were explained to the parents/guardian and a written informed consent was obtained from them. Oral hygiene instructions, diet counseling, were given during the period of study.

-Evaluation was done for the following:

1. Clinical parameter: OHI-S Index:

An explorer was passed across the buccal surface of incisal one-third, two- third and cervical region of the index teeth i.e 16,11,26,36,41,46. Each of the six teeth was given a score from 0-3 on the basis of the Simplified Oral Hygiene Index (OHI-S) (23).

2. Method of dental plaque sample collection:

Plaque samples were collected from each patient between 9 to 10 AM using a sterile wooden toothpick in scraping motion from buccal and lingual surfaces from occlusal to gingival one-third from the index teeth i.e 16,11,26,36,31,46. The collected samples were stored in an eppendorf tube (5ML) containing 3ml of saline solution and immediately transferred to the laboratory.

3. Method of saliva sample collection:

For saliva collection, the child was asked to drool out saliva in a sterile container and 2- 3ml of unstimulated whole saliva was collected. The containers were labelled and stored in an ice box set below 4oC and sent immediately for *S. mutans* analysis.

-Method of Varnish Application:

Prior to application of varnish, oral prophylaxis was carried out to standardize the oral cavity with the lowest possible amount of biofilm. Isolation was done with cotton rolls & saliva ejector and teeth were dried with gentle blow of air for 30 s using three way air syringe. Approximately 0.1 ml of the designated varnish was applied with an applicator tip quadrant-wise sequentially starting from the upper arch to all the teeth.

Post application, subjects were instructed not to rinse their mouth, not to drink or eat anything for 3 hours, and not to brush till the next day. A non-fluoridated tooth paste was also provided to be used during the study period.

-Laboratory Phase:

Evaluation of *S. mutans* in both plaque and saliva samples were done by 3 methods

A. CULTURE TEST:

Mitis Salivarius Bacitricin Agar (MSB Agar) was prepared for multiplication and selection of *Streptococcus mutans* on the agar plates. The saliva samples were diluted 1:1 and plaque samples were diluted 1:2 before spreading on to the agar plates for colony counting. 8 sections were made on the petriplate and the colonies observed in 1/8 section was counted and then multiplied by 8 to get the no. of colonies in the whole plate. Then the no. of colonies obtained is multiplied by the dilution factor to get total no. of colonies in 1 ml saliva or plaque – CFU/ml. The *S.mutans* colonies were identified by its typical morphology (frosted glass appearance) raised, convex, undulate, opaque, pale-blue colonies that are granular.

B. OPTICAL DENSITY (OD):

Optical density (OD) was obtained at 600 nm with the help of a Spectrophotometer (Spectronic 20).This technique is use for monitoring growth kinetic which is based on light scattering technique

C. PCR PROCEDURE:

In the present study, 60 saliva and 60 plaque samples were randomly selected and subjected to PCR (15 from each group).

The bacterial culture is subjected to SDS (sodium dodecyl sulphate). DNA molecules that are released from the disrupted bacterial cells are precipitated out using Ethanol.

PCR study was done using primers GTFB- F5’ACTACACTTTCGGGTGGCTTGG and GTFB- R5’CAGTATAAGCGCCAGTTTCATC. Denaturation was done at 95°C for 30 s, which was followed by annealing at 59°C for 30 s, and extension at 72°C for 1 min. This amplification was repeated for 33 cycles. The final extension cycle was run at 72°C for 5 min. PCR products were subjected to electrophoresis on 1.5% agarose gel and stained with ethidium bromide

The clinical and laboratory parameters were assessed at baseline (T=0), followed by one month (T=1) and 3 months (T=3) after the application of varnish

**Figure 2:**
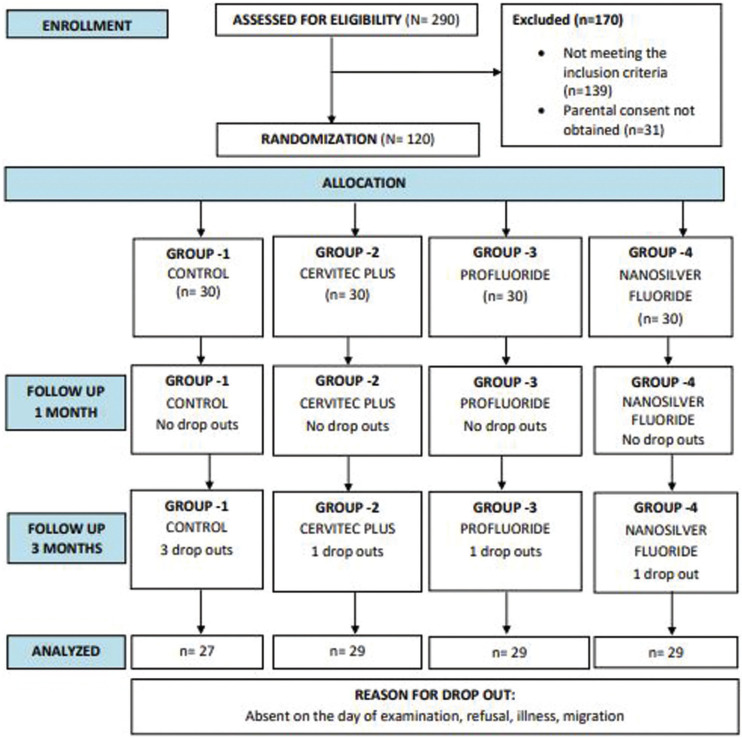
CONSORT flow diagram.

The final study sample comprised of 114 children with 6 dropouts at 3 months (Fig. 2).

## Results

One way ANOVA followed by post-hoc bonferroni test were used to compare the *S. mutans* count at different time intervals following the use of different varnishes.

After 6 subjects drop out from the initial sample of 120 subjects during the follow-up period, the study was continued on only 114 children, aged 8-10 years, comprising of 63 girls (55.26%) and 51 boys (44.75%) which formed the final study sample at the end of 3 months.

OHI-S: The mean baseline OHI-S for all the groups was in the range 0.424±0.138 to 0.504± 0.169. Maximum reduction in OHI-S score was observed in group II at the end of 3 months which was 0.12±0.15, the change in OHI-S was statistically significant in only group II (Table 1).

**Table 1:**
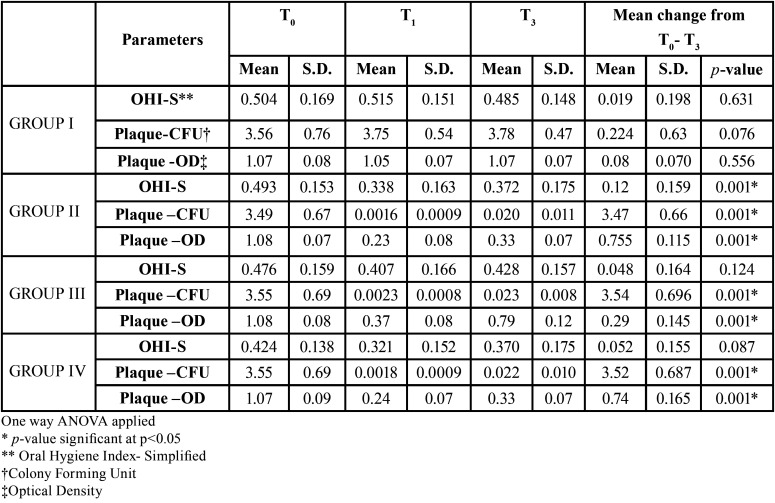
Table showing mean OHI-S, Plaque CFU and OD values recorded for Group I, II, III and IV at baseline (T0), 1 month (T1) and 3 months (T3).

PLAQUE: The mean baseline plaque CFU for all the groups was in the range of 3.49±0.67 CFU to 3.56± 0.76 CFU. Maximum reduction in plaque CFU score 1 month after varnish application was observed in group II followed by group IV, group III and group I. After 3 months also maximum reduction was found in group II. The reduction was statistically significant in all the groups except the control group. Similar result was also seen on evaluation by optical density (Table 1).

The intergroup comparison showed that there was no statistically significant difference in plaque CFU score amongst group II, III and IV at the end of 3 months. Whereas when OD was compared statistically significant difference was found between group I and all other groups. Highly significant difference was seen in group II and group III and also in group III and IV. Whereas, the difference amongst group II and IV was not found to be significantly different.

SALIVA: The mean baseline salivary CFU for all the groups was in the range 3.27±0.79 CFU to 3.45± 0.74CFU. Statistically significant difference in the *S.mutans* values was seen both by culture method and OD in all the three experimental groups at the end of 3 month. The maximum reduction was seen in both group II and group IV after 1 month. After 3 months maximum reduction was found in group II (Table 2). The intergroup comparison did not show any significant difference between group II, III and IV at the end of 3 months (Table 3).

**Table 2:**
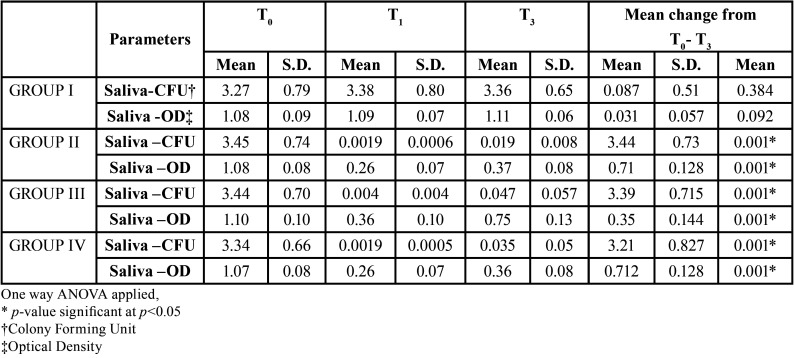
Table showing mean Saliva CFU and OD values recorded for Group I, II, III and IV at baseline (T0), 1 month (T1) and 3 months (T3).

**Table 3:**
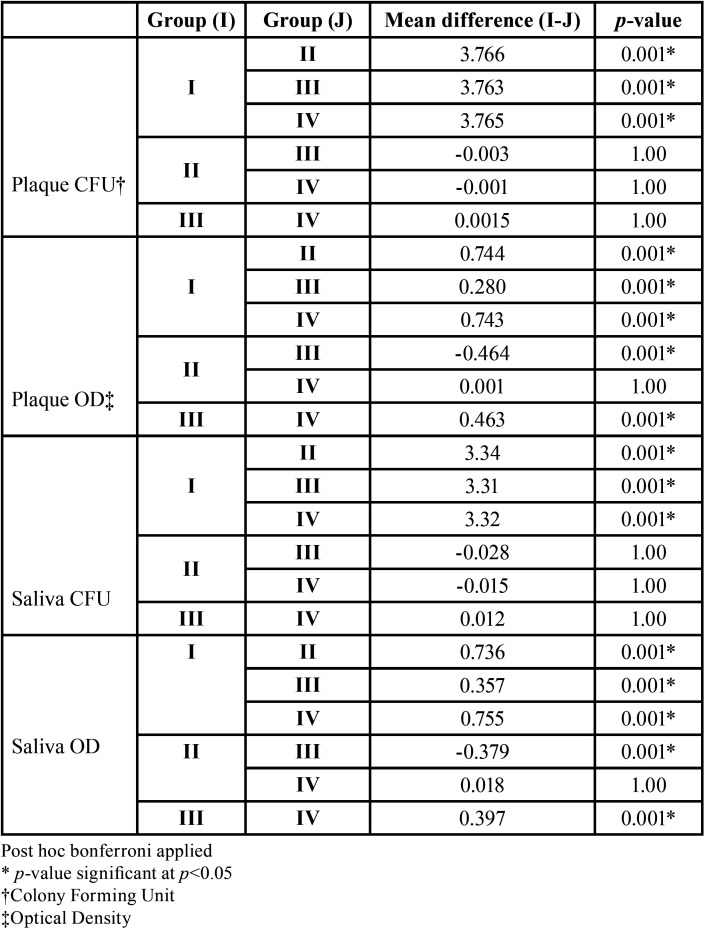
Intergroup comparison of CFU & OD among the four groups at 3 month.

Evaluation by OD showed highly significant difference between group II and III and between group III and IV whereas; there was a non-significant difference amongst group II and IV (Table 3).

PCR: The frequency of identification of *S.mutans* was more in plaque samples when compared with saliva samples at baseline and after 3 months. Post varnish application, the frequency of identification of *S.mutans* decreased in group II and group IV more as compared to other groups in both plaque and saliva samples but it was statistically not significant (Table 4).

**Table 4:**
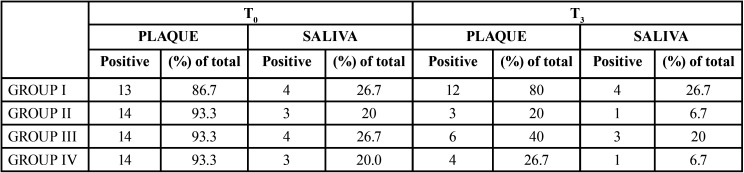
Table showing frequency of identification of S mutans in plaque and saliva samples using PCR at baseline (T0) and 3 months (T3) for Group I, II, III and IV.

## Discussion

The result of the present study has shown that significant reduction of *S. mutans* was seen after 3 months, in both saliva and plaque, when a single application of sodium fluoride, chlorhexidine and NanoSilver Fluoride varnish was done in caries free children.

Jiang Q *et al*. reported that, S mutans is present not only in caries active but also in caries free subjects (24). It was seen that children who were caries free at first visit became caries active within few period of time (25) suggesting that if intervention is done only during caries active period, it will be difficult to prevent new cases of dental caries. Thus caries free children were chosen in order to assess the actual antimicrobial effect of different varnishes on the *S.mutans*.

 In this study, a non-intensive application regime of varnish application of once at baseline was chosen which was in accordance with the study done by Aljaradi *et al*. 2013, Ben khadra 2019 (26,27). Although, Twetman *et al*. in their study had a more intensive application regime, it was observed that intensive application did not extend the effect of the varnish (28). In the present study, a single application regime was followed by a significant reduction of S mutans even after 1 month. This effect is called “BURST EFFECT” which is due to the release of active agents from the varnish (29).

Nano silver fluoride is a new varnish containing nano silver particles with fluoride and chitosan as a stabilizing agent. The silver nanoparticles form free radicals and damage the bacterial cell membrane and make it porous leading to cell death. Furthermore, silver ions can interact with sulfuryl groups during protein synthesis and interfere in the replication of DNA (30). The fluoride in NSF imparts it with remineralizing properties (31) which reduces biofilm formation and adhesion. Therefore NSF has a synergistic effect of being antimicrobial as well as remineralizing agent.

Agnihotri *et al*., Morones JR *et al*., Espinosa *et al*. demonstrated that smaller the size of silver nanoparticles, greater is its activity (19,32,33). This is mainly due to better contact with the surface of the bacteria (19). In the present study, the nanoparticle size of silver was 8 ± 2.0 nm which was able to show antimicrobial properties comparable to that of chlorhexidine though in an *in vitro* study, Besinis A *et al*. in 2014 had shown that the antibacterial activity of AgNPs was 25 fold higher than chlorhexidine when 56.8 ± 18 nm sized AgNPs were used (17).

The fluoride content in NSF is shown to be 10,147 ppm (15) as compared with 22,600 for NaF varnish (34), yet, NaF varnish showed lesser reduction of *S.mutans* when compared to NSF. This shows that incorporation of silver nano particles to sodium fluoride greatly enhances its antimicrobial properties.

## Conclusions

A statistically significant reduction in *S.mutans* counts of both plaque and saliva was seen after the application of Nano Silver Fluoride, sodium fluoride & chlorhexidine varnishes. Since the reduction by NSF was comparable to NaF and CHX, it can be proposed that NSF varnish is more advantageous than CHX and NaF as it has dual properties of acting as an antibacterial as well as a remineralizing agent.

Further long term studies should be conducted to ascertain the role of NSF varnish in the management of children having caries.
